# Complete genome sequence of *Archaeoglobus profundus* type strain (AV18^T^)

**DOI:** 10.4056/sigs.942153

**Published:** 2010-06-15

**Authors:** Mathias von Jan, Alla Lapidus, Tijana Glavina Del Rio, Alex Copeland, Hope Tice, Jan-Fang Cheng, Susan Lucas, Feng Chen, Matt Nolan, Lynne Goodwin, Cliff Han, Sam Pitluck, Konstantinos Liolios, Natalia Ivanova, Konstantinos Mavromatis, Galina Ovchinnikova, Olga Chertkov, Amrita Pati, Amy Chen, Krishna Palaniappan, Miriam Land, Loren Hauser, Yun-Juan Chang, Cynthia D. Jeffries, Elizabeth Saunders, Thomas Brettin, John C. Detter, Patrick Chain, Konrad Eichinger, Harald Huber, Stefan Spring, Manfred Rohde, Markus Göker, Reinhard Wirth, Tanja Woyke, Jim Bristow, Jonathan A. Eisen, Victor Markowitz, Philip Hugenholtz, Nikos C Kyrpides, Hans-Peter Klenk

**Affiliations:** 1 DSMZ - German Collection of Microorganisms and Cell Cultures GmbH, Braunschweig,; Germany; 2 DOE Joint Genome Institute, Walnut Creek, California, USA; 3 Los Alamos National Laboratory, Bioscience Division, Los Alamos, New Mexico, USA; 4 Biological Data Management and Technology Center, Lawrence Berkeley National Laboratory, Berkeley, California, USA; 5 Oak Ridge National Laboratory, Oak Ridge, Tennessee, USA; 6 University of Regensburg, Microbiology – Archaeenzentrum, Regensburg, Germany; 7 HZI – Helmholtz Centre for Infection Research, Braunschweig, Germany; 8 University of California Davis Genome Center, Davis, California, USA; * Corresponding author: Hans-Peter Klenk

**Keywords:** hyperthermophilic, marine, strictly anaerobic, sulfate respiration, hydrogen utilization, hydrothermal systems, *Archaeoglobaceae*, GEBA

## Abstract

*Archaeoglobus profundus* (Burggraf *et al*. 1990) is a hyperthermophilic archaeon in the euryarchaeal class *Archaeoglobi*, which is currently represented by the single family *Archaeoglobaceae*, containing six validly named species and two strains ascribed to the genus '*Geoglobus*' which is taxonomically challenged as the corresponding type species has no validly published name. All members were isolated from marine hydrothermal habitats and are obligate anaerobes. Here we describe the features of the organism, together with the complete genome sequence and annotation. This is the second completed genome sequence of a member of the class *Archaeoglobi*. The 1,563,423 bp genome with its 1,858 protein-coding and 52 RNA genes is a part of the *** G****enomic* *** E****ncyclopedia of* *** B****acteria and* *** A****rchaea * project.

## Introduction

Strain AV18^T^ (= DSM 5631 = JCM 9629 = NBRC 100127) is the type strain of the species *Archaeoglobus profundus* [1,2]. It is the second of five species currently ascribed to the genus *Archaeoglobus*, of which the type species is *A. fulgidus*, described in 1988 [3]. Strains for all *Archaeoglobus* species were isolated from marine hydrothermal systems, yet *A. fulgidus* originates from a shallow marine hydrothermal system at Volcano, Italy [3] whereas *A. profundus* was isolated from a deep sea hot vent area (depth: 2000 m) at Guaymas, Mexico [1]. The genome sequence of the type strain from a third species of the *Archaeoglobaceae* – *Ferroglobus placidus* [4] – has been completed very recently (Feb 2010) at the Joint Genome Institute (CP001899). Here we present a summary classification and a set of features for *A. profundus* strain AV18^T^, together with the description of the complete genomic sequencing and annotation.

## Classification and features

Six species with validly published names and two strains ascribed to the not invalidly published genus '*Geoglobus*'  [5,6] are currently assigned to the *Archaeoglobi*, all of which were isolated from marine hydrothermal systems ranging from shallow water to deep sea habitats of 4,100 m depth. Five species thereof are accounted to the genus *Archaeoglobus*: *A. profundus*, *A. fulgidus*, *A. veneficus* [7], *A. infectus* [8] and *A. solfaticallidus* [9]. Publications about the taxonomy of the *Archaeoglobi* often mention another species of this genus (“*A. lithotrophicus*”) isolated from deep oil reservoirs [10], but no formal species description has been published, therefore this ninth species is excluded from comparisons shown in this work.

Based on 16S rRNA gene sequences, the closest related type strain is *F. placidus* [4] with 96.5% sequence identity, while the other type strains of the genus *Archaeoglobus* share 91.9-95.0% sequence identity [11], with the non validly published ‘*Geoglobus*’ strains inbetween (94.4%). The nearest related genera are *Pyrococci* and *Thermococci* with about 86% sequence identity. Searching the NCBI non-redundant nucleotide database with the 16S rRNA sequence of *A. profundus*, 73 sequences of at least 90% sequence identity were found. Fifty of these sequences belong to uncultured archaeal phylotypes from environmental samples, all others were identified as belonging to the *Archaeoglobaceae*. These samples originated from marine hydrothermal systems at the Mid-Atlantic Ridge [12,13] and AJ969472, the East Pacific Rise [14,15], Izu-Bonin Arc [16], and Southern Mariana Trough (AB293221, AB293225, AB293242, AB293237) in the Western Pacific Ocean, Iheya Basin (Okinawa Trough) in the East China Sea [17,18], the Gulf of California [19,20], a seafloor borehole at Juan de Fuca Ridge in the Pacific Ocean [21], from high temperature oil reservoirs [22], and from terrestrial hot springs in Europe [23], North America [24-26], East Asia (FJ638514, FJ638518-23 FJ638504, FJ638508) and Southeast Asia [27]. These numerous findings (as of January 2010) corroborate and extend the early assumption [1] that members of the *Archaeoglobaceae* may be widely distributed across hydrothermal habitats.

**Figure 1:**
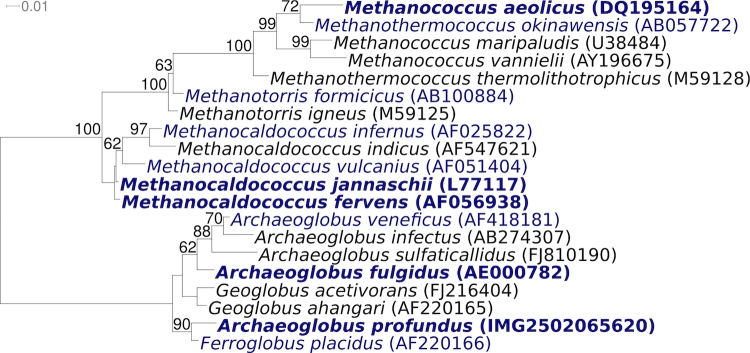
Phylogenetic tree highlighting the position of *A. profundus* AV18^T^ relative to the other type strains within the family. The tree was inferred from 1,334 aligned characters [28,29] of the 16S rRNA gene sequence under the maximum likelihood criterion [30] and rooted in accordance with a current taxonomy [31]. The branches are scaled in terms of the expected number of substitutions per site. Numbers above branches are support values from 1,000 bootstrap replicates if larger than 60%. Lineages with type strain genome sequencing projects registered in GOLD [32] are shown in blue, published genomes in bold: *Methanococcus aeolicus* (CP000743), *Methanocaldococcus fervens* (CP001696), *Methanocaldococcus jannaschii* [33] and *A. fulgidus* [34], two of the very first organisms whose genome sequences have been revealed.

Figure 1 shows the phylogenetic neighborhood of *A. profundus* AV18^T^ in a 16S rRNA based maximum likelihood [35] phylogenetic tree, which is in agreement with earlier inferences of the phylogeny of this taxon [5,6,8,9,31]. Remarkably, *A. profundus* clusters together with *F. placidus*, apart from the cluster containing the other three species of the genus *Archaeoglobus*, indicating polyphyly of the genus and therefore possibly the need for taxonomic emendation, as discussed previously [9].The sequence of the single 16S rRNA gene copy in the genome of *A. profundus* AV18^T^ is identical with the previously published 16S rRNA gene sequence derived from DSM 5631 (AJ299219), which contained five ambiguous base calls.

**Figure 2:**
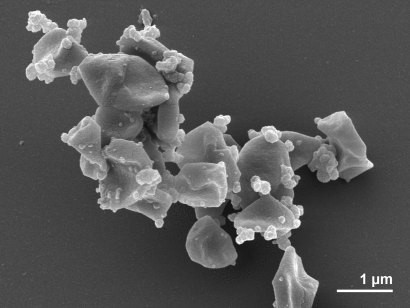
Scanning electron micrograph of cells of *A. profundus* strain AV18^T^

Cells of *A. profundus* AV18^T^ are reported as Gram stain-negative, highly irregular cocci, occurring singly or in pairs (Figure 2 and Table 1) [1]. They have dimensions of approximately 0.7-1.3 µm x 1.4-1.9 µm. The organism shows a blue-green fluorescence at 420 nm UV light, indicating the presence of coenzyme F_420_, and contains a cell envelope composed of subunits covering the membrane, which is visible in thin sections [1]. Motility and flagella were not observed [1,43] in contrast to all other members of this genus, with the exception of *A. sulfaticallidus*, which was described very recently [9].

**Table 1:** Classification and general features of *A. profundus* strain AV18^T^ according to the MIGS recommendations [36]

**MIGS ID**	**Property**	**Term**	**Evidence code**
	Classification	Domain *Archaea*	TAS [38]
		Phylum *Euryarchaeota*	TAS [39]
		Class *Archaeoglobi*	TAS [40]
		Order *Archaeoglobales*	TAS [41]
		Family *Archaeoglobaceae*	TAS [99]
		Genus *Archaeoglobus*	TAS [3]
		Species *Archaeoglobus profundus*	TAS [1]
		Type strain AV18	TAS [1]
	Gram stain	negative	TAS [1]
	Cell shape	coccoid, highly irregular	TAS [1]
	Motility	not motile	TAS [1]
	Sporulation	nonsporulating	NAS
	Temperature range	65-90°C	TAS [1]
	Optimum temperature	82°C	TAS [1]
	Salinity	>9-36 g/l (optimum 18 g/l)	TAS [1]
MIGS-22	Oxygen requirement	obligate anaerobic	TAS [1]
	Carbon source	acetate, pyruvate, lactate, yeast extract, meat extract, peptone, acetate containing crude oil	TAS [1]
	Energy source	H_2_	TAS [1]
MIGS-6	Habitat	deep sea hydrothermal system	TAS [1]
MIGS-15	Biotic relationship	free-living	NAS
MIGS-14	Pathogenicity	none	TAS [100]
	Biosafety level	1	TAS [100]
	Isolation	cores of hot sediment	TAS [1]
MIGS-4	Geographic location	Guaymas, Mexico	TAS [1]
MIGS-5	Sample collection time	before or around 1989	NAS
MIGS-4.1 MIGS-4.2	Latitude Longitude	14.84 -17.23	NAS
MIGS-4.3	Depth	-2,000 m	TAS [1]
MIGS-4.4	Altitude	-2,000 m	TAS [1]

Evidence codes - IDA: Inferred from Direct Assay (first time in publication); TAS: Traceable Author Statement (i. e. a direct report exists in the literature); NAS: Non-traceable Author Statement (i. e. not directly observed for the living, isolated sample, but based on a generally accepted property for the species, or anecdotal evidence). These evidence codes are from the Gene Ontology project [42]. If the evidence code is IDA, then the property was directly observed for a living isolate by one of the authors or an expert mentioned in the acknowledgements.

Growth of strain AV18^T^ occurs between 65 and 90°C with an optimum at 82°C, at a pH ranging from 4.5 to 7.5 and a concentration of NaCl between 0.9 and 3.6% [1]. *A. profundus* is mixotrophic under strictly anaerobic conditions [1] with hydrogen as an essential energy source and sulfate, thiosulfate and sulfite as electron acceptors, producing H_2_S [1].

All members of the genus *Archaeoglobus* can utilize hydrogen as electron donor, in addition, *A. fulgidus*, *A. veneficus* and *A. solfaticallidus* can use at least a subset of the organic compounds pyruvate, formate, acetate or lactate [9,43]. Electron acceptors are those of *A. profundus* (see above) except for *A. veneficus* and *A. infectus*) which are incapable of utilizing sulfate [9,43]. Carbon sources can be CO_2_ (except for *A. profundus* and *A. infectus*) or organic compounds [8,9,43]. Due to differences mainly in metabolism, a new genus was introduced for *F. placidus* [4]: Unlike previously described *Archaeoglobales*, *F. placidus* is capable of growing by nitrogen reduction, and oxidation of ferrous iron or sulfide, but unable to reduce sulfate [4]. Besides, it is the only reported case of an archaeon which can anaerobically oxidize aromatic compounds, by reduction of Fe(III) [37]. Other published species of this class are “*Geoglobus ahangari*” [5] and the recently reported “*G. acetivorans*” [6]. The genus “*Geoglobus*” again separates from the other *Archaeoglobaceae* by characteristic metabolic features: in cultivation experiments, the sole electron acceptor used by these species is Fe(III) and they are reported to be the first hyperthermophilic organisms exhibiting growth upon anaerobic oxidation of long chain fatty acids [5,6].

### Chemotaxonomy

In *A. profundus*, acyclic C_40_ tetraether, an unknown compound at an R_f_ in the range of cyclized glycerol-dialkyl-glycerol tetraethers, and a C_20_:C_20_ diether constitute the membrane core lipids, whereas C_20_:C_25_ diethers are absent, similar to *A. fulgidus* [1]. However, *A. profundus* differs from *A. fulgidus* in the composition of complex lipids, consisting of two phosphoglycolipids at R_f_ 0.10 and 0.13, and four glycolipids at R_f_ 0.40, 0.45, 0.60, 0.65, while the latter contains two phosphoglycolipids at R_f_ 0.10 and 0.215, one phospholipid at R_f_ 0.30 and one glycolipid at R_f_ 0.60 [1]. The cell envelope consists of an S-layer and is rifampicin and streptolydigin resistant [1].

## Genome sequencing and annotation

### Genome project history

This organism was selected for sequencing on the basis of its phylogenetic position [44], and is part of the *** G****enomic* *** E****ncyclopedia of* *** B****acteria and* *** A****rchaea * project [45]. The genome project is deposited in the Genomes OnLine Database [32] and the complete genome sequence is available in GenBank. Sequencing, finishing and annotation were performed by the DOE Joint Genome Institute (JGI). A summary of the project information is shown in Table 2.

**Table 2:** Genome sequencing project information

**MIGS ID**	**Property**	**Term**
MIGS-31	Finishing quality	Finished
MIGS-28	Libraries used	Three 454 pyrosequence libraries, standard and two paired end (8 kb and 15kb insert sizes) and one Illumina library (300bp inset size)
MIGS-29	Sequencing platforms	454 Titanium, Illumina
MIGS-31.2	Sequencing coverage	136× 454 Titanium, 30× Illumina GAii
MIGS-30	Assemblers	Newbler, phrap
MIGS-32	Gene calling method	Prodigal, GenePRIMP
	INSDC ID	CP001857 (chromosome) CP001858 (plasmid)
	GenBank Date of Release	January 20, 2010
	GOLD ID	Gc01188
	NCBI project ID	32583
	Database: IMG-GEBA	2501939633
MIGS-13	Source material identifier	DSM 5631
	Project relevance	Tree of Life, GEBA

### Growth conditions and DNA isolation

*A. profundus* AV18^T^, DSM 5631, was grown anaerobically in DSMZ medium 519 (*A. profundus* medium) [46] at 85°C. DNA was isolated from 1-1.5 g of cell paste using Masterpure Gram-positive DNA purification kit (Epicentre) with a modified protocol for cell lysis, st/DL according to Wu *et al*. [45].

### Genome sequencing and assembly

The genome of strain AV18^T^ was sequenced using a combination of 454 and Illumina sequencing platforms. All general aspects of library construction and sequencing can be found at http://www.jgi.doe.gov/. Pyrosequencing reads were assembled using the Newbler assembler version 2.0.0-PostRelease-10/28/2008 (Roche). Possible misassemblies were corrected with Dupfinisher [47] or transposon bombing of bridging clones (Epicentre Biotechnologies, Madison, WI). Gaps between contigs were closed by editing in Consed, by custom primer walk or PCR amplification. A total of 26 finishing reads were produced to close gaps, to resolve repetitive regions, and to raise the quality of the finished sequence. Illumina reads were used to improve the final consensus quality using an in-house developed tool (the Polisher, unpublished). The error rate of the completed genome sequence is less than 1 in 100,000. Pyrosequence provided 136× coverage of the genome and the final assembly contains 718,930 454-pyrosequence reads.

### Genome annotation

Genes were identified using Prodigal [48] as part of the Oak Ridge National Laboratory genome annotation pipeline, followed by a round of manual curation using the JGI GenePRIMP pipeline [49]. The predicted CDSs were translated and used to search the National Center for Biotechnology Information (NCBI) nonredundant database, UniProt, TIGR-Fam, Pfam, PRIAM, KEGG, COG, and InterPro databases. Additional gene prediction analysis and functional annotation was performed within the Integrated Microbial Genomes - Expert Review (IMG-ER) platform [50].

## Genome properties

**Figure 3:**
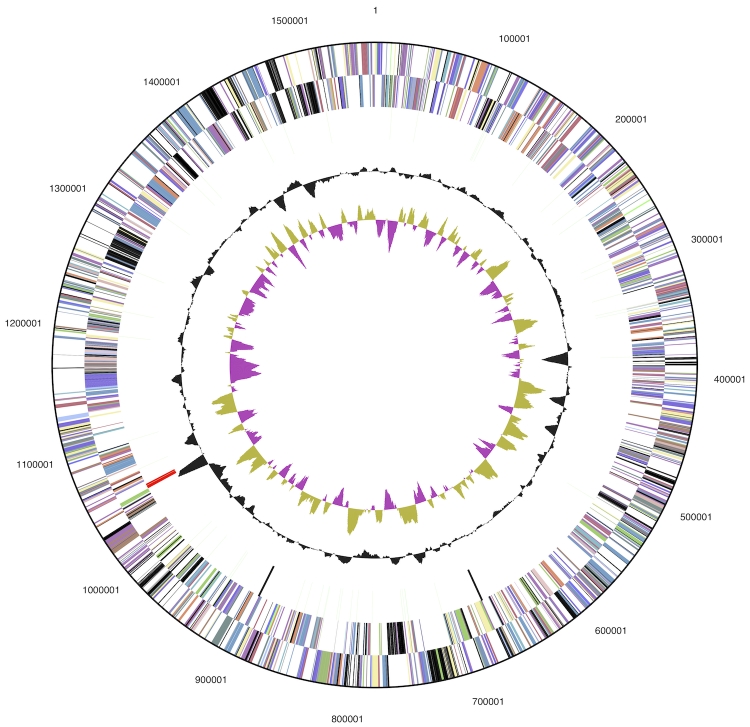
Graphical circular map of the genome (without the 2.8 kbp plasmid). From outside to the center: Genes on forward strand (color by COG categories), Genes on reverse strand (color by COG categories), RNA genes (tRNAs green, rRNAs red, other RNAs black), GC content, GC skew.

The 1,563,423 bp genome consists of a 1,560,622 bp chromosome and a 2,801 bp plasmid with an overall G+C content of 42.0% (Table 3 and Figure 3). Of the 1,909 genes predicted, 1,858 are protein-coding genes, and 52 RNAs; 35 pseudogenes were also identified. The majority of the protein-coding genes (60.0%) were assigned a putative function while the remaining ones were annotated as hypothetical proteins. The distribution of genes into COGs functional categories is presented in Table 4.

**Table 3:** Genome Statistics

**Attribute**	**Value**	**% of Total**
Genome size (bp)	1,563,423	100.00%
DNA coding region (bp)	1,474,996	94.34%
DNA G+C content (bp)	656,709	42.00%
Number of replicons	2	
Extrachromosomal elements	1	
Total genes	1,909	100.00%
RNA genes	52	2.67%
rRNA operons	1	
Protein-coding genes	1,858	97.33%
Pseudo genes	35	1.83%
Genes with function prediction	1,145	59.98%
Genes in paralog clusters	167	8.75%
Genes assigned to COGs	1,267	66.37%
Genes assigned Pfam domains	1,301	68.15%
Genes with signal peptides	141	7.39%
Genes with transmembrane helices	328	17.18%
CRISPR repeats	0	

**Table 4:** Number of genes associated with the general COG functional categories

**Code**	**value**	**%age**	**Description**
J	150	8.1	Translation, ribosomal structure and biogenesis
A	2	0.1	RNA processing and modification
K	67	3.6	Transcription
L	79	4.3	Replication, recombination and repair
B	4	0.2	Chromatin structure and dynamics
D	13	0.7	Cell cycle control, cell division, chromosome partitioning
Y	0	0.0	Nuclear structure
V	4	0.2	Defense mechanisms
T	46	2.5	Signal transduction mechanisms
M	40	2.2	Cell wall/membrane biogenesis
N	18	1.0	Cell motility
Z	0	0.0	Cytoskeleton
W	0	0.0	Extracellular structures
U	21	1.1	Intracellular trafficking and secretion
O	53	2.9	Posttranslational modification, protein turnover, chaperones
C	109	5.9	Energy production and conversion
G	41	2.2	Carbohydrate transport and metabolism
E	110	5.9	Amino acid transport and metabolism
F	47	2.5	Nucleotide transport and metabolism
H	85	4.6	Coenzyme transport and metabolism
I	24	1.3	Lipid transport and metabolism
P	57	3.1	Inorganic ion transport and metabolism
Q	7	0.4	Secondary metabolites biosynthesis, transport and catabolism
R	201	10.8	General function prediction only
S	169	9.1	Function unknown
-	642	34.6	Not in COGs

## Insights from the genome sequence

### Replicons

*A. profundus* AV18^T^ is the second type strain of the *Archaeoglobi* with a fully sequenced genome to be published [34]. In contrast to *A. fulgidus*, the genome of AV18^T^ has a small cryptic plasmid of 2,801 bp that contains four genes which appear to have no other function than the maintenance of this replicative unit. It displays a slightly lower G+C content (40%) than the rest of the genome and is negatively supercoiled, as demonstrated for pGS5 by López-García *et al*. [51]. The version of the plasmid presented here differs in three positions from the sequence of pGS5, resulting in one split gene.

### Origin of replication

Unlike in bacteria, the archaeal initiation of the replication fork can occur at more than one site (origin of replication, ORI) on the chromosome [59], which heuristics used for bacteria fail to locate. Likewise, the ORI in *A. profundus* could not be detected by the use of Ori-Finder (http://tubic.tju.edu.cn/Ori-Finder/) [52], which is consistent with several attempts to discover the replication origin in *A. fulgidus* by such methods [53-57]. Well-conserved replication signature patterns are known from both *Crenarchaea* and *Euryarchaea* [59]. In the genome of *A. fulgidus*, two almost identical ORB elements of 22 bases length are located at 65.6% of the length of the genome, which is in agreement with the position of the (single) ORI of this organism, identified by experimental origin mapping [58].

Pattern searching in the non-coding regions of the genome sequence of AV18^T^ revealed a situation very much comparable to that of *A. fulgidus*: Two identical, but inverted ORB elements (**T**TTCCACAGGAAA**T**AAAGGGG**T**) were identified between genes Arcpr_1540 and Arcpr_1543; with 1,264 bases of distance (containing two hypothetical proteins) between each other, differing in only two bases from either ORB element in *A. fulgidus*. This position marks the predicted origin of replication in *A. profundus*, which is likewise far away from the (single copy of) *cdc 6 *(generally considered as marker gene for the ORI) Arcpr_0001, located at the very beginning of the chromosome sequence. The presence of further active chromosomal ORIs cannot be excluded, but the strong similarity to the situation in *A. fulgidus* suggests that the genome of AV18^T^ also contains only one origin of replication.

### Shine-Dalgarno sequences

Before the start of the translation process, the recruitment of a ribosome to the mRNA is mediated by a species-specific DNA motif, the Shine-Dalgarno (SD) sequence [60], constituting the ribosome binding site (RBS) closely upstream of the coding region. In order to identify the SD consensus sequence in *A. profundus*, the Pattern Discovery Tool (oligo-analysis) of RSAT (http://rsat.ulb.ac.be/rsat/) [61] was used for *de-novo* motif discovery within 50 bp regions upstream of all protein-coding genes in the genome of AV18^T^, with a background model estimated from its whole genome nucleotide sequence. The most frequently detected heptanucleotide was GGAGGTG, matching the complementary sequence one base shifted from the 3'-end of the 16S rRNA: TCTGCGGCTGGAT**CACCTCC**T-3' (bold: matching sequence)  is obviously involved in ribosome recruitment.

Using Prodoric Virtual Footprint software [62], the frequencies of heptanucleotides which are able to match (allowing one mismatch) the 3'-end of the 16S rRNA were determined. A significant drop was observed when the seven base window reached the base C at position eleven of the reverse complement 16S rRNA terminus (Table 5), indicating that interactions with the RBS are restricted to the ten most distal bases.

**Table 5:** Reverse complement of the 16S rRNA 3'-end

**scanning** **heptanucleotide**	**abs. frequency**	**rel. frequency**
AGGAGGT	555	29.9%
GGAGGTG	569	30.6%
GAGGTGA	565	30.4%
AGGTGAT	549	30.0%
GGTGATC	283	15.2%
GTGATCC	88	4.7%
TGATCCA	89	4.8%
GATCCAG	53	2.9%
ATCCAGC	58	3.1%
TCCAGCC	45	2.4%

Assessment of the frequencies of heptanucleotide sections with one acceptable mismatch in 50 bp regions upstram of all genes in AV18^T^.

In total, the upstream regions of 950 genes match at least one of the four most frequently observed heptanucleotides, representing 51% of all protein-coding genes. Bakke *et al.* [63] recently evaluated three current genome annotation pipelines on the basis of the *Halorhabdus utahensis* genome [64] and recommended the integration of species-specific SD-motifs into the ORF-calling process of automated genome annotation pipelines, in order to determine the correct start codons of protein-coding genes. In several members of the *Archaea* (group A *sensu* Torarinsson *et al.* [65]), however, the benefits of this approach might be limited by the fact that single genes and first genes of operons are often leaderless (in *A. fulgidus*: 50%), thus containing no SD sequence [65]. Despite the expected abundance of leaderless transcripts, the percentage of genes preceded by SD sequences is significantly higher than the percentage observed in the genome of *H. utahensis* [64] based on the same annotation pipeline: Scanning 50 bp areas upstream of all *H. utahensis* genes with the most common heptanucleotide (allowing one mismatch) matched in only 8.6% of the respective areas of all genes, while the genome of strain AV18^T^ reached 30.6%.

The heptanucleotide matching the very end of the 16S rRNA terminus is slightly less represented than the following shifted motifs, indicating that the final T of the 16S terminus might not be as essential for the RBS recognition as the preceding bases. This is consistent with recent insight into crystal structure and dynamics of the SD helix in an initiation-like 70S ribosome complex of *Thermus thermophilus*, showing base pairings of positions two to nine from the 3'-end of the 16S rRNA and the SD sequence of the mRNA, excluding an interaction with the very last base of the rRNA [66]. Transferring these results to the analysis of the SD sequence in strain AV18^T^, the comparatively high observed frequency of motif AGGAGGT is likely due to the setting of the motif scan, which allows one mismatch. The same is true for the opposite side of the SD sequence, and the reason for the high frequency of motif AGGTGAT. Therefore, the predicted complete, species-specific consensus RBS motif of *A. profundus* is the 8-base pattern GGAGGTGA, which represents the functional sequence area of interaction in the initial contact between ribosome and mRNA in *A. profundus.*

### tRNAs and Codon usage

By the use of tRNAscan-SE [67], a total of 48 tRNAs were identified and the coverage of all possible codons was assessed. Two codons are redundantly represented by tRNAs: AUC (two copies of Ile-tRNA gene) and AUG (four copies of Met-tRNA gene). None of the codons ending on U are are present. Apart from these, AUA is the only codon that is not directly associated with a tRNA. The translation of this codon is strictly dependent on wobble modifications that are carried out by different modification systems in the three domains of life. Insight into the archaeal mechanism of AUG translation was gained very recently [68], involving the polyamine-conjugated modified base 2-agmatinylcytidine (agm^2^C) at the wobble position of the corresponding tRNA, and the enzyme tRNA^Ile^-agm^2^C synthetase (TiaS), which catalyzes the agm^2^C formation using agmatine and ATP. A candidate for this enzyme in *A. profundus* AV18^T^ is Arcpr_0572, identified by sequence similarity with the experimentally confirmed TiaS gene in *A. fulgidus* (AF2259). Arcpr_0572 displays the highest similarity to AF2004, one of three genes belonging to the same gene family in *A. fulgidus*. Therefore, a bidirectional best BLAST hit to the experimentally confirmed TiaS gene in *A. fulgidus* cannot be identified in *A. profundus*.

Redundant or missing representation of codons by tRNAs has apparently no effect on the frequency of codon usage (determined by program gp_cusage, http://www.bioinformatics.org/ genpak/; data not shown), as both are used in some cases more frequently, in other cases less frequently than the corresponding alternative codon which is allocated exactly one tRNA. The tRNAs for Trp, Tyr and one of the Met-tRNAs contain introns of 60, 17 and 26 bases length, respectively. Concerning the frequencies of the utilized start codons, 84.6% of the protein-coding genes start with AUG, while the frequency of this start codon in *A. fulgidus* is considerably lower (76.5%). The frequency of the alternative start codon GUG (10.4%) in *A. fulgidus* is almost twice as high (19.5%), reflecting the difference in GC-content (*A. fulgidus*: 48.6%), while UUG is rare in both (*A. profundus*: 4.4%, *A. fulgidus*: 3.2%). The correct prediction of start codons plays a decisive role in the ORF-calling process. In a comparison between three current genome annotation pipelines, 90% of the predicted genes shared the same stop codons, while only 48% thereof agreed in start codon prediction, resulting in different gene lengths [63]. The average gene length in the genome of AV18^T^ is only 773 bp, while *A. fulgidus* genes are on average 815 bp long, a difference which – along with the different frequencies of alternative start codons – might also be caused by the different annotation pipelines used for both genomes [63].

### Comparative genomics

The genome sequencing for the type strain of another species of the *Archaeoglobaceae*, *F. placidus* AEDII12DO^T^, provided the opportunity for a genome-wide comparative analysis among three species of the *Archaeoglobaceae*. All of these analyses were performed using IMG online tools [69] with the default settings, unless stated otherwise. Metabolic pathways were reconstructed by the combination of online resources such as NCBI (http://www.ncbi.nlm.nih.gov/), KEGG (http://www.genome.jp/kegg/ [70], ), BRENDA [71] and MetaCyc [72]. Orthology of genes was determined by bidirectional best BLAST [73] hits and the comparison of functional groups using EBI InterProScan [74]. Phylogenetic comparisons are restricted to validly named species only. This limitation excludes e.g. ‘*Nanoarchaeum equitans*’, ‘*Cenarchaeum symbiosum*’ and strains assigned to the category *Candidatus*.

**Figure 4:**
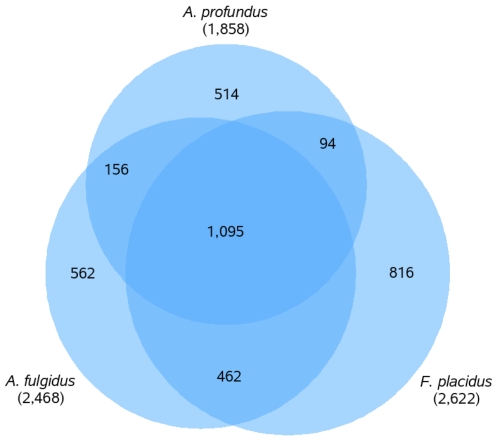
Venn-diagram depicting the intersections of protein sets (total numbers in parentheses) of the three completely sequenced *Archaeoglobi* genomes. All intersections concerning *A. profundus* are gene counts of AV18^T^, the remaining intersection between *A. fulgidus* and *F. placidus* only, are gene counts in *A. fulgidus*. Due to variable copy numbers of several genes in the three species, the fragments do not add up to the total numbers of genes for *A. fulgidus* and *F. placidus*.

The genome size of *A. profundus* AV18^T^ (1.6 Mb) is significantly smaller than those of *A. fulgidus* (2.2 Mb, 2,468 protein-coding genes [34]) and *F. placidus* (2.2 Mb, 2,622 protein-coding genes). Figure 4 shows the numbers of shared genes in a Venn-diagram. *A. fulgidus* and *F. placidus* share a considerable number of genes that are not present in *A. profundus*. These genes are associated with a wide range of functions and pathways, some of which will be discussed below in more detail. This fraction of genes includes the seven subunits of carbon monoxide dehydrogenase, two of the key enzymes for the β-oxidation of fatty acids, and genes belonging to the CRISPR/Cas system.

The genome of strain AV18^T^ contains only a small percentage (8.7%) of paralogous genes, as compared to 12.8% in *F. placidus* and 17.1% *A. fulgidus* (http://img.jgi.doe.gov). Likewise, the percentage of genes with signal peptides in strain AV18^T^ (7.4%) is considerably lower than those of *A. fulgidus* (10.8%) and *F. placidus* (10.1%.

### DNA-polymerase genes

To date, four distinct DNA-dependent DNA-polymerase families are known. They are specifically distributed across the three domains of life, with the unrelated B and D family polymerases being present in *Archaea* [75]., The evolutionary divergence further discriminates *Crenarchaeota*, which have up to three family B monomeric DNA polymerases, and *Euryarchaeota*, which generally have one monomeric family B DNA polymerase and one heterodimeric family D DNA polymerase [76].

Three different family B DNA polymerases have been detected in Archaea [77-79], B3 being the single family B DNA-polymerase identified in the genome of *A. profundus* AV18^T^. The respective gene, Arcpr_0273 is also present in the genomes of *A. fulgidus* (in contrast to the current annotation, which assigned subtype B1 to this gene) and *F. placidus*. Each of the three *Archaeoglobi* contains also one copy of the euryarchaeal family D DNA polymerase, and *A. fulgidus* is unique by having a second family B DNA polymerase gene (AF0693), belonging to subtype B2.

### RNA polymerase B subunit

The DNA-dependent RNA polymerase (RNAP) subunit B was previously reported as a suitable tool for phylogenetic reconstructions [80]. A split in the B subunit of the RNA polymerase – resulting in the fragments B' and B'' –  has been reported for a subset of the euryarchaeal branch containing the methanogens and halophiles, based on the first five available archaeal sequences of this gene. This split has been described to be phylogenetically conserved and its use for supporting or refuting branching topologies has been suggested [80].

**Figure 5:**
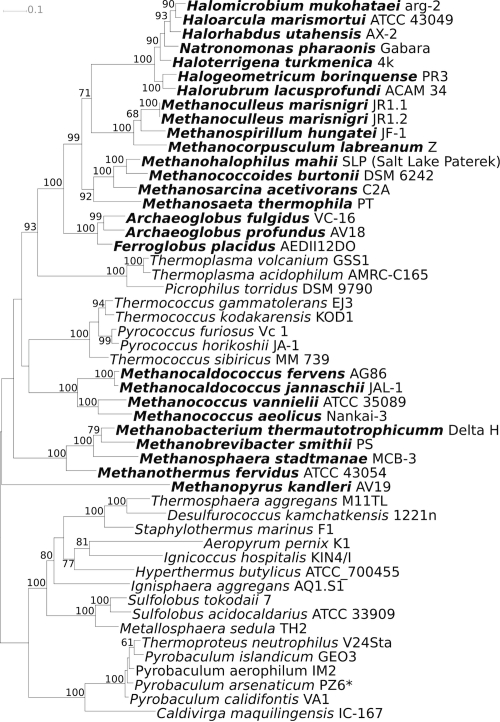
Phylogenetic tree of archaeal type strains with fully sequenced genomes, inferred using the maximum likelihood criterion [30], based on an alignment of the RNA polymerase B subunit sequence and rooted with the node which separates *Cren*- and *Euryarchaeota*. The alignment was inferred by Muscle [81] software, using the PROTCATLGF substitution model. Bootstrapping was performed using RAxML [30] and values above 60% mark the corresponding nodes. Species containing a conserved split in the RNA polymerase B subunit gene are displayed in bold.

Here, the validity of this observation was reassessed, based on a larger number of available archaeal RNAP subunit B genes (n=77) from all of the currently available fully sequenced genomes. For organisms exhibiting the above mentioned split, the corresponding amino acid sequences of the B' and B'' component were joined and a phylogenetic tree was inferred (Figure 5), showing clusters that are largely consistent with the 16S rRNA tree topology [31]. The topology of this tree suggests a polyphyletic origin of the split in the B subunit, however, the best tree under the constraint of monophyly is not significantly worse (α=0.01) than the tree shown [30]. Therefore, this tree is not significantly in conflict with the assumption of a unique origin of the split into the B' and B'' components of RNAP. Further mapping of the species exhibiting the conserved split against the 16S rRNA phylogeny confirmed the suggestion that this split is the result of a singular event which had taken place in the evolution of the *Euryarchaea* [80]. The lowest branching family containing this conserved split are *Archaeoglobaceae* represented by *A. profundus*, *A. fulgidus* and *F. placidus* (genes: Arcpr_0976/7, AF1886/7, Ferp_0762/3). Likewise, all taxa which diverged later from the main branch, i.e. *Methanococci*, *Methanobacteria*, *Methanomicrobia*, *Halobacteria* and possibly *Methanopyrus kandleri* (the basal position of the latter in the 16S rRNA-based phylogenetic tree is disputed [82]), contain this split without exception. Taxa which diverged earlier (*Thermococci*, *Thermoplasmata* and all *Crenarchaeota*) have the unfragmented version of the B subunit, equally without exception among validly named organisms.

### CRISPRs

Clusters of Regularly Interspaced Short Palindromic Repeats (CRISPRs) represent a recently discovered prokaryotic defense system against viral attacks [83,84]. Although frequently observed in members of the *Archaea* (~90%), *A. profundus* completely lacks any CRISPRs. In contrast, the genome of *A. fulgidus* contains three large CRISPR spacer/repeat arrays, consisting of 44 to 60 repeats of lengths between 30 and 37 bases per repeat [34]. *Ferroglobus* contains twelve CRISPR arrays of variable repeat lengths and copy numbers (JGI, unpublished).

### Motility and chemotaxis genes

A widespread phenomenon among *Archaea* and *Bacteria* is their ability to sense environmental conditions by the chemotaxis system and actively move towards more favorable locations by the activity of the flagellum. The archaeal flagella are non-homologous to those of *Bacteria*, and their components are encoded by one or two well-conserved gene clusters (*fla* clusters) [85], which have been subject to extensive phylogenetic studies [86]. *A. profundus* is reported to be non-motile [1,43], showing no flagellation, in contrast to most *Archaeoglobi*, including *A. fulgidus* [3] and *F. placidus* [4]. Unexpectedly, the genome sequence revealed the presence of a complete *fla* gene cluster (Arcpr_1384 – Arcpr_1391) and the preflagellin peptidase *FlaK* gene (Arcpr_0277), [85]. The situation in *A. fulgidus* (AF_1048–AF_1055, *flaK*-gene: AF_0936) and *F. placidus* (Ferp_1456–Ferp_1463, *flaK*-gene: Ferp_0061) is virtually identical in content, order and orientation of genes of the *fla* cluster, therefore the different phenotypes are unexpected. However, a conflict between presence of the flagella genes and the phenotypically observed lack of motility is not unique for *A. profundus*, but has also been reported for *Methanosarcina* species [86]. Also the reverse, even more surprising case – observed motility, but lacking homologues of the genes coding for flagellum components – has been reported for *Pyrobaculum aerophilum* and *M. kandleri* [86]. Some of our electron micrograph images (data not shown) displayed structures which might be flagella on few *A. profundus* cells, mainly observed in larger cell clots. This indicates that *A. profundus* might be flagellated under certain conditions, not necessarily for motility reasons, but also functions such as cell-cell adhesion to form cell aggregates (as reported for *Methanosarcinales*) are thinkable. In any case, the possibility of artifacts (e.g. the presence of fragments from damaged cells) causing the observed structures on our electron micrographs cannot be excluded.

Unlike the flagellum genes, the archaeal chemotaxis system is homologous to the one in bacteria (for a review see [87]). Using the IMG Phylogenetic Profiler, the genomes of *A. profundus*, *A. fulgidus* and *F. placidus* revealed the same genetic components for a chemotaxis system (AF1034, AF1037–AF1042, AF1044; Arcpr_1371–Arcpr_1376, Arcpr_1378, Arcpr_1379; Ferp_1072–Ferp_1377, Ferp_1379, Ferp_1990), with the only exception that *A. fulgidus* displays two copies of the methyl-accepting chemotaxis protein, while the others only have one. This observation again supports the hypothesis that *A. profundus* might be motile under certain conditions, otherwise not only its flagellum-genes, but also the genetic components for chemotaxis would remain unused. However, the archaeal system of motility and chemotaxis is not yet fully unraveled. Especially the proteins constituting the flagellar motor and the link between chemotactic signal transduction and the motility apparatus [88]. The lack of undescribed essential components for this complex cannot be ruled out for *A. profundus*, which might be the reason for the observed immobility.

### β-oxidation of long-chain fatty acids

The ability of the *Archaeoglobi* to anaerobically oxidize long-chain fatty acids has been discussed controversially: although a β-oxidation system in *A. fulgidus* was predicted from the genome sequence [34], followed by reports of growth on crude and olive oil [89], “*G. ahangari”* was later reported to be the first hyperthermophile with this capacity [5]. Very recently, *A. fulgidus* VC-16 was demonstrated to be capable of growth on a wide range of fatty acids and alkenes as sole source of energy, using thiosulfate or sulfate as the electron acceptor [90]. Likewise, the genome of *F. placidus* contains at least the four key enzymes for β-oxidation, suggesting the presence of this pathway

In the first description of *A. profundus*, minor growth on acetate containing crude oil was observed [1]. With the here reported complete genome sequence, it becomes clear that this organism is unique within its sequenced relatives in lacking two of the four key enzymes for β-oxidation: 3-hydroxyacetyl-CoA-dehydrogenase (EC:1.1.1.35) and enoyl-CoA-hydratase (EC:4.2.1.17). Therefore it can now be posited that the reported growth on crude oil was most likely due to the contained traces of acetate, as the organism lacks essential components required for the oxidation of long-chain fatty acids *via* β-oxidation.

### Nitrate reduction

Currently, *F. placidus* is the only validly named member of the *Archaeoglobi* which has been shown to be able to use nitrate as electron acceptor. A cluster of genes encoding a putative nitrate reductase has yet been identified in *A. fulgidus* (AF0173–AF0176) and discussed in the literature [34,91], again resulting in a conflict between genetic equipment and observed metabolic features, as a biochemical evidence for nitrate reduction is still missing in *A. fulgidus*. Homologues of these genes are also present in *A. profundus*, though distributed in two separate locations in the genome (Arcpr0672, Arcpr0674, Arcpr1727, Arcpr1728) and in *F. placidus* (Ferp_0121-Ferp_0124). The latter contains another nitrate reductase gene cluster (Ferp_0311–Ferp_0314, additional gamma subunit: Ferp_1088), which might be the reason for the observed nitrate respiration in culture conditions, while specificity and activity of the more widely distributed hypothetical nitrate reductase gene cluster remains subject to further experiments.

### Sulfate reduction

The reduction of sulfurous compounds is the central electron accepting pathway in the metabolism of *A. profundus*. The genetic equipment for the catalysis of the corresponding reactions is largely equivalent to the one previously described for *Desulfovibrio* species and postulated for *Desulfohalobium retbaense* [92]. The respective genes of *A. profundus* have been determined by sequence comparisons and identification of the corresponding functional groups. A notable difference to the mechanism of sulfate-reduction in *Desulfovibrio* species is the absence of a periplasmic cytochrome buffer composed of cytochrome *c_3_*.

Thus, genes encoding a molybdopterin oxidoreductase MOP complex – as described for *Desulfovibrio desulfuricans G20* [93] – have not been identified in the genome of *A. profundus*. The MOP complex is thought to transfer electrons to menaquinone by interacting with periplasmic reduced cytochrome *c_3_*. The regeneration of the reduced menaquinone pool is most likely performed by a set of F_420_-nonreducing hydrogenase family proteins (genes: Arcpr_1002, Arcpr_1005 and Arcpr_1006) which transfer electrons originating from the oxidation of hydrogen – *via* a co-localized gene (Arcpr_1004) encoding a membrane associated cytochrome b – to oxidized menaquinone molecules in the membrane. Another option for the reduction of the menaquinone pool is given by a F_420_H_2_:quinone oxidoreductase complex, utilizing electrons supplied by F_420_H_2_. This reduced electron carrier originates from the pathway of reverse methanogenesis, which is a typical feature of the *Archaeoglobi*. The F_420_H_2_:quinone oxidoreductase complex has been studied in *A. fulgidus* [94,95] and a similar gene cluster exists in *A. profundus* (Arcpr_1575–Arcpr_1584). One of three additional proteins which have been found in the purified complex of *A. fulgidus* [94] has also been identified in *A. profundus* (Arcpr_0247) by reciprocal BLAST search.

The quinone-interacting membrane-bound oxidoreductase (QMO)-complex (Arcpr_0661–Arcpr_0663) transfers electrons *via* the heterodimeric AprAB complex (Arcpr_1261, Arcpr_1262) from the reduced menaquinone pool in the membrane to activated sulfate (APS, adenosine-5'-phosphosulfate), forming sulfite. Likewise, the membrane-associated DsrMKJOP (Arcpr_1727–Arcpr_1731) complex transfers electrons from the same source to the dissimilatory sulfite reductase (Arcpr_0139-Arcpr_0141), catalyzing the reduction from sulfite to sulfide. Both processes are used to generate a membrane potential with the major purpose of ATP production.

### Carbon monoxide dehydrogenase

The enzymatic equipment used for reverse methanogenesis in *A. fulgidus* is equivalent to the the “Eastern branch” of the Wood-Ljungdahl pathway, which is also present in acetogenic organisms [96]. This pathway consists of two branches, each reducing a CO_2_ into a methyl- and a carbonyl-moiety, respectively, which are joined forming acetyl-CoA. This metabolic capacity is not present in *A. profundus*, due to a blocked “Western branch” (acetyl-CoA decarbonylase/synthase is absent), a fact which has been discovered already in 1995 [97]. The consequence for *A. profundus* is its inability to grow autotrophically [43,97]. In both *A. fulgidus* and *F. placidus*, all genes for the complete set of seven different subunits of the acetyl-CoA decarbonylase/synthase are present and both can grow autotrophically, like all other described *Archaeoglobaceae*, except *A. profundus* and *A. infectus*. However, *A. profundus* might be able to use the presence of the Eastern branch of the Wood-Ljungdahl pathway for a certain amount of CO_2_-fixation, as the intermediate 5,10-methylene-tetrahydro-methanopterine can be branched off to other pathways, e. g. by formaldehyde-activating enzyme (Arcpr_1052) into formaldehyde, or by glycine hydroxymethyltransferase (Arcpr_0687, Arcpr_1587) to the glycine, serine and threonine metabolism.

Besides providing comprehensive insight into the genetic equipment, the completely sequenced genome of *A. profundus* revealed instances in which the presence of certain genes suggests capabilities which were not observed in laboratory cultivation, such as flagellation or chemotaxis. Reasons for this might be paralogous genes, *e. g*. having altered, yet unidentified substrate specificity, defect genes, pseudogenes or genes which are permanently transcriptionally deactivated, as reported for hydrogenase genes in *Methanosarcina acetivorans* [98]. Alternatively, the biochemic capacities might only be exhibited under specific unknown environmental conditions, which are yet to be reproduced in laboratory experiments.
